# Advancements in analytical methods for studying the human gut microbiome

**DOI:** 10.14440/jbm.2024.0050

**Published:** 2024-11-18

**Authors:** Gijsbert J. Jansen, Gerard P. Schouten, Marit Wiersma

**Affiliations:** NL-Lab, Biotrack, Leeuwarden, Friesland, 8912 AP Netherlands

**Keywords:** Culturing, Fluorescence *in situ* hybridization, Gut microbiome, Next-generation sequencing, Polymerase chain reaction

## Abstract

**Background:**

The human gut microbiome, a complex ecosystem of microorganisms, plays a crucial role in maintaining human health. Perturbations in its composition are linked to a wide range of health conditions.

**Analytical techniques:**

Researchers employ various techniques to study the gut microbiome, each having its own strengths and limitations. Polymerase chain reaction (PCR) is highly sensitive but dependent on the quality of DNA extraction. Next-generation sequencing (NGS) is powerful but can be costly and requires extensive data analysis. Furthermore, the accuracy of NGS results also depends heavily on the quality of the DNA extraction process. Culture methods, while useful, are biased and time-consuming. Fluorescence *in situ* hybridization (FISH) excels in visualizing specific microbial populations and is the only method capable of providing *in situ* information. However, until recently, FISH was heavily reliant on human interpretation of digital photomicrographs, limiting its application in high-throughput strategies. Additionally, the sensitivity of FISH is restricted by the number of cells visualized.

**Conclusion:**

Understanding the strengths and weaknesses of these methods is essential for drawing robust conclusions in microbiome research.

## 1. Introduction

The human gut microbiome constitutes a complex and dynamic ecosystem of microorganisms, encompassing bacteria, archaea, viruses, and fungi, which coexist in a finely-tuned equilibrium. This microbial assembly orchestrates essential functions such as nutrient digestion, absorption, immune regulation, and even neurological processes.^1^ Recent research has underscored the profound repercussions of perturbations in the gut microbiome composition, as they are associated with a wide spectrum of health conditions, including obesity,^2^ inflammatory bowel diseases,^3^ and mental health disorders.^4^ These revelations have intensified interest in understanding the gut microbiome’s profound impact on human health and have driven the concomitant development of analytical methodologies. Advanced methods to study the gut microbiome are therefore essential for advancing research in the field of nutrition and medicine.

To investigate the gut microbiome, researchers employ a diverse array of techniques, each offering unique insights into this complex microbial landscape. This article provided a comparative discussion of the most relevant performance characteristics of methods currently used in the field of gut microbiome analysis. Only primary methods, those that can be applied directly to stool samples, are considered, *i.e*., culturing, polymerase chain reaction (PCR), next-generation sequencing (NGS), and fluorescence *in situ* hybridization (FISH). Therefore, MALDI-TOF is excluded from this comparison, as it requires pre-culturing and is considered a secondary method. Historically, selective culture methods have enabled the isolation and identification of specific bacterial strains, providing foundational insights into the diversity of gut microbiota. The selective culture of bacteria from fecal samples is a critical diagnostic tool in microbiology. This method involves applying fecal matter to selective media designed to foster the growth of specific bacterial populations while inhibiting others. This is achieved through the incorporation of certain substances, such as antibiotics, bile salts, or specific nutrient compositions.^5^

PCR techniques, on the other hand, empower the amplification and quantification of specific DNA sequences, enabling the detection of individual microbial taxa.^6^ PCR applied to bacteria from stool samples is a molecular technique that amplifies target DNA sequences, facilitating the identification of bacterial species present in the fecal matter. The process begins with the extraction of DNA from the stool sample. Primers—short DNA fragments complementary to target bacterial sequences—are then added. Through thermal cycling, DNA polymerase enzymes amplify these target sequences exponentially. The PCR results may be further analyzed in two ways: (i) By normalizing the relative expression of the bacteria against the total amount of bacteria present,^7^ or (ii) by Sanger sequencing. For the first option, the PCR must consist of a universal eubacteria primer and primers specific to the 16S rRNA of the bacteria that are going to be measured.^7^ For the second option, the PCR only contains one primer and fluorescently-labeled di-deoxynucleotide triphosphates, which terminate the elongation of the DNA strand, generating DNA fragments of varying lengths. These fragments are separated by DNA sequencing machines by size and give chromatograms as output, thereby giving sequence reads.^8^

The advent of NGS as a successor of the Sanger sequencing has revolutionized the field. NGS methods such as 16S rRNA gene sequencing and shotgun metagenomics permit the simultaneous analysis of thousands of microbial species and their genetic potential. NGS of bacterial 16S rRNA genes from stool involves high-throughput sequencing to analyze the genetic material of bacteria present in fecal samples. This method starts with the extraction of total DNA/RNA, followed by amplification of the 16S rRNA gene, a region containing enough sequence variability to allow for specific identification. NGS allows for the parallel sequencing of millions of DNA fragments, including the amplified 16S rRNA genes. By comparing these sequences to known bacterial databases, the composition and diversity of the bacterial community in the stool can be determined, providing insights into gut health and disease.^9^,^10^

Furthermore, FISH studies have provided the capability to visualize specific microbial cells within the gut microbiome and allow for the *ex vivo* quantitative analysis of bacterial communities in the stool. FISH employs fluorescently-labeled probes that specifically bind to the 16S rRNA sequences of bacteria present in stool. This method involves fixation and permeabilization of bacteria, allowing probe entry, followed by hybridization with the fluorescently-labeled probes. Upon binding, the probes illuminate under a fluorescence microscope, enabling the visualization and identification of specific bacterial populations within the complex fecal microbiota.^11^

PCR, NGS, and FISH all use the 16S rRNA gene for bacterial determination. The 16S rRNA gene consists of nine highly-conserved and nine hypervariable regions that play a pivotal role in microbial identification and phylogenetic studies. Its utility stems from its presence across nearly all prokaryotes, which, combined with its slow evolutionary rate, makes the 16S rRNA an ideal universal marker for distinguishing between different bacterial species. However, it is important to note that, in most applications, only specific hypervariable regions of the 16S rRNA gene are targeted, rather than the entire gene. These variable regions, interspersed between the conserved sequences, allow for species-level discrimination while minimizing the challenges associated with utilizing the full-length gene. The selective amplification and analysis of these regions enhance both the efficiency and accuracy of microbial community profiling techniques. In the case of taxonomic resolution, there is no principle difference between the methods that utilize 16S rRNA as the analytical substrate (PCR, NGS, FISH). However, the taxonomic resolution of culture-based tests is significantly lower than the taxonomic resolution of PCR, NGS, and FISH.^12^,^13^

The development and integration of these analytical methods have facilitated an in-depth exploration of the gut microbiome’s diversity and functionality. As a result, 16S rRNA gene sequencing has unraveled the correlation between reduced microbial diversity and conditions such as inflammatory bowel disease, while shotgun metagenomics has identified potential microbial markers for obesity and diabetes.^14^ In parallel, FISH studies have exposed the spatial organization of the microbiome within the gut mucosa, delivering invaluable insights into its role in health and disease.^15^ Despite the remarkable progress in gut microbiome research, it is imperative to recognize that there exists a considerable knowledge gap, particularly when it comes to a comprehensive comparative analysis of the aforementioned analytical methods in the context of gut microbiome research. Understanding the strengths and limitations of each technique is paramount for refining experimental designs and drawing robust conclusions.

## 2. Strengths and limitations of the analytical methods

Performance parameters, such as detection limits, repeatability, and sensitivity (Table 1), are vital for researchers assessing the suitability and reliability of each method for their specific study needs. Additionally, time metrics—such as time to result and hands-on time (Table 1)—provide additional practical considerations, as they can significantly impact the efficiency and workflow of research projects.

**Table 1:** Overview of performance parameters of the methods applied mostly in gut microbiome research

Technology	Principle	Detection	Repeatability	Sensitivity	TTR	HoT
Culturing	Active cell division on (specific) medium	10^2^ CFU/g^23^; 10^4^ CFU/g^24^; 10^4^ – 10^5^ CFU/g^25^	0.22 – 0.4711; 0.39626	84 – 90%27; 65%28; 82 – 90%^28^	≥72 h^27^; 3 days^29^	10 min27; 3h27
PCR	Enzymatic amplification of specific gene (s)	1.5×10^3^ n/g^30^; 1.0×10^3^ n/g^31^; 1.0×10^5^ n/g^32^	>0.90^31^; 0.4 – 0.833; 0.97^34^	68 – 85%^25^; 94 – 98%^27^	1.5 – 4.5 h^25^; 4.5 h^27^; <20 h29; >2 h31	20 – 40 min^25^; 10 min^27^; 10 – 20 min^31^
NGS	Enzymatic amplification of the genome leading to a collection of fragmented DNA sequences (library) followed by sequencing	1×10^6^/read^35^	0.8536; 0.38 – 0.93^37^	>90%^9^	>8 h^9^	10 – 30 min^17^
FISH	Specific hybridization of naturally present ribosomal RNA	1.0×10^9^/g38; 1.0×10^7^/g39; 1.0×10^6^/g^40^	0.07 – 0.1411; 30 – 74%^41^	100%27; 97%29; 95%^42^	45 min27; <20 h^29^	10 min^27^

Notes: All fragments derived from the initial PCR amplification for NGS.

Abbreviations: CFU/g: Colony forming unit per gram; n/g: Cell number per gram; FISH: Fluorescence *in situ* hybridization; HoT: Hands-on time (for one sample); NGS: Next-generation sequencing; PCR: Polymerase chain reaction; TTR: Time to result.

While PCR is reasonably sensitive and specific, it has limitations in terms of taxonomic resolution or granularity, meaning it may not provide a full taxonomic classification of microorganisms at the species or strain level. Primer bias can impact the accuracy of the results and lead to incomplete or inaccurate microbial profiles. Effective DNA extraction from gut samples can be challenging due to the presence of inhibitors, like complex polysaccharides and bile salts.^16^ The PCR’s strength lies in its targeted approach, which can also be a limitation. It is not ideal for discovering novel or unexpected microorganisms in the gut, as it, like FISH, requires prior knowledge of specific genes or sequences of interest. Furthermore, while widely used, PCR can be costly in terms of reagents and equipment, particularly in high-throughput studies involving many samples.^17^ Additionally, PCR analysis can be labor-intensive, requiring skilled personnel to perform and interpret the results.

NGS is also a resource-intensive method. The costs associated with library preparation, sequencing, and data analysis can be prohibitive for some research projects. Additionally, NGS generates vast amounts of data, requiring expertise in handling of large datasets. The need for specialized software and computational resources can pose a barrier for some researchers. Despite its ability to provide a comprehensive view of the gut microbiome, NGS can be affected by sampling bias. The DNA extraction process may favor certain microbial species or result in the underrepresentation of specific taxa, leading to an incomplete picture of the microbiome.^18^ Many NGS platforms produce short-read sequences. While these are useful for taxonomic profiling and gene presence/absence analysis, they may not capture the full genetic diversity of the gut microbiome, including strain-level differences and the identification of novel species. Finally, NGS typically has longer turnaround times compared to PCR-based methods. Sample processing, library preparation, sequencing, and data analysis can be as fast as 8 hours but often take several days or even weeks, which may not be suitable for research projects with urgent timelines.^19^

Alternatively, culture methods are heavily biased towards microorganisms that are cultivable on specific growth media. The gut microbiota consists largely of fastidious or anaerobic bacteria, which makes them difficult to cultivate. This bias can lead to a skewed representation of the microbiome, resulting in omission of a significant portion of its diversity. Culture methods typically require several days to weeks for microbial growth and colony identification. This extended time frame can hinder real-time or urgent research needs, particularly in clinical settings where rapid identification of pathogens is critical. The majority of gut microbiota are non-culturable, rendering this approach unsuitable for studying a comprehensive microbiome. Moreover, when microorganisms are isolated and cultured outside their natural environment, they may lose certain functions or properties that are crucial for understanding their role within the gut ecosystem. This limitation can hinder the accurate representation of their functional contributions.^20^

Finally, FISH typically targets specific RNA (rRNA or mRNA) sequences to identify microorganisms. Similar to PCR-based methods, the choice of probes may introduce bias, as FISH only detects microorganisms that match the probe’s sequence. However, FISH is well-suited for visualizing specific microbial populations in their *in vivo* habitus, which is a unique methodological characteristic of this technique.^21^ Historically, preparing samples for FISH has been labor-intensive and requires technical expertise. Fortunately, fully autonomous systems for sample preparation are available and operational in laboratories worldwide. Also, FISH can now identify the “trinity” of microbiological detection, *i.e*., cell identity, cell number, and microbial activity (expressed as the ribosomal content per cell) in one assay. Interpreting FISH results can be challenging, especially in complex microbial communities. However, recent advancements in artificial intelligence (AI), particularly neural networks, have enabled objective analysis and interpretation of microscopic FISH images. Furthermore, FISH is now easily scalable for high-throughput studies, making it much more suitable for large-scale gut microbiome research projects.^22^

## 3. Conclusion

While PCR, NGS, and FISH are valuable tools for gut microbiome research, it’s essential to be aware of their limitations and potential biases. For example, PCR is more appropriate for targeted investigations rather than comprehensive and unbiased profiling of the gut microbiome. Although NGS is a powerful tool for gut microbiome analysis, it comes with several challenges and limitations, particularly in terms of cost, data analysis complexity, and potential biases. Culture methods, while historically important in microbiology, also have significant limitations in studying the gut microbiome. Their bias towards culturable microorganisms, limited taxonomic resolution, and time-consuming nature make them less suitable for comprehensive analyses, especially when compared to modern molecular and sequencing-based methods. The FISH, on the other hand, is a valuable method for *in situ* visualization and identification of specific microbial populations, including both cell numbers and proteogenic activity in the gut. This method is particularly effective when combined with algorithms for AI that interpret microscopic FISH images. However, the sensitivity of FISH is restricted by the number of cells that can be visualized. Overall, modern forms of FISH, which utilize robotized sample preparation and AI-driven image interpretation, demonstrate strong sensitivity, scalability, and efficient hands-on time. These advantages suggest that they may become the next industry standard in microbiome research.

Although PCR and NGS are currently the most common methods for gut microbiome research, the utilization of automated FISH in combination with AI-driven interpretation of fluorescent images has the potential to revolutionize microbial research. Advancements in machine learning will enable high-throughput and precise identification of microbial populations. Artificial intelligence algorithms will improve image segmentation, classification, and quantification, leading to faster and more accurate analyses of microbial diversity and activity. This integration of automated FISH and AI may offer significant insights into the gut microbiome’s role in health, disease, and therapeutic interventions.

## Data Availability

Not applicable.
